# Evaluation of Lateral and Vertical Dimensions of Micromolds Fabricated by a PolyJet™ Printer

**DOI:** 10.3390/mi12030302

**Published:** 2021-03-13

**Authors:** Sindhu Vijayan, Pravien Parthiban, Michinao Hashimoto

**Affiliations:** 1 Pillar of Engineering Product Development, Singapore University of Technology and Design, 8 Somapah Road, Singapore 487372, Singapore; sindhu_vijayan@mymail.sutd.edu.sg (S.V.); pravien_parthiban@outlook.com (P.P.); 2 Digital Manufacturing and Design Centre, Singapore University of Technology and Design, 8 Somapah Road, Singapore 487372, Singapore

**Keywords:** microfluidics, PolyJet 3D printing, fidelity of 3D printing

## Abstract

PolyJet™ 3D printers have been widely used for the fabrication of microfluidic molds to replicate castable resins due to the ease to create microstructures with smooth surfaces. However, the microstructures fabricated by PolyJet printers do not accurately match with those defined by the computer-aided design (CAD) drawing. While the reflow and spreading of the resin before photopolymerization are known to increase the lateral dimension (width) of the printed structures, the influence of resin spreading on the vertical dimension (height) has not been fully investigated. In this work, we characterized the deviations in both lateral and vertical dimensions of the microstructures printed by PolyJet printers. The width of the printed structures was always larger than the designed width due to the spreading of resin. Importantly, the microstructures designed with narrow widths failed to reproduce the intended heights of the structures. Our study revealed that there existed a threshold width (*w_d_*′) required to achieve the designed height, and the layer thickness (a parameter set by the printer) influenced the threshold width. The thresholds width to achieve the designed height was found to be 300, 300, and 500 μm for the print layer thicknesses of 16, 28, and 36 μm, respectively. We further developed two general mathematical models for the regions above and below this threshold width. Our models represented the experimental data with an accuracy of more than 96% for the two different regions. We validated our models against the experimental data and the maximum deviation was found to be <4.5%. Our experimental findings and model framework should be useful for the design and fabrication of microstructures using PolyJet printers, which can be replicated to form microfluidic devices.

## 1. Introduction

This paper describes the characterization of polymer-jet (PolyJet™) 3D printers for their ability to produce microscale structures. Previous studies reported that the CAD drawing and the microstructures printed by PolyJet printers differ in the widths due to the spreading of the photoresin [1,2,3]. However, the effect of the spreading on the height of the printed structures has not been investigated. In this research, we studied the capability of PolyJet printers to print microstructures in terms of width (*w_p_*) and height (*h_p_*). We have experimentally studied the deviation in dimensions and developed mathematical models to explore the dynamics of printing. Models were developed for two different regions considering the dynamics of printing in the low and high widths. The experimental findings suggested that the spreading of the resin contributed to not only increasing the width of the features but also decreasing the height of the same features when the designed width of the features was below particular thresholds. These findings explain the inherent limitation of the printer to produce microstructures with accurate dimensions. The models can serve as a tool to predict the outcomes of PolyJet printing. The knowledge obtained in our study set out guidelines to obtain the designed microscale features by PolyJet printing, which shall be of interest to the microfluidic community for the fabrication of the micromolds.

### 1.1. Microfluidics

Microfluidics is the science and technology of fluids in small scales, with dimensions in the range of tens to hundreds of micrometers. Microfluidic systems offer advantages such as the requirement of low volumes, high sensitivity, fast response, small footprints, and precise control over the experimental parameters [4]. Such advantages can be exploited in a wide range of applications including chemical synthesis, biochemical analysis, drug delivery, detection, and sensing [5,6,7,8,9]. Microfluidic devices to perform such operations are initially fabricated by photolithography [10] and machining [11]. Those devices are fragile and found to be unsuitable for handling fluids. The advent of novel polymeric materials in the latter half of the 20^th^ century resulted in the production of materials suitable for the microfabrication of devices. Microfabrication demanded polymers with desired properties such as low glass transition temperature, non-hygroscopic nature, chemical and mechanical stability, optical transparency, and flexibility. A method of fabrication collectively termed soft lithography was adopted to use such polymers for microfabrication [12,13,14].

### 1.2. Soft Lithography and 3D Printing

Soft lithography has been widely adopted for the fabrication of microfluidic devices. Soft lithography involves replica molding to transfer the features of the micromold to castable elastomeric resins and other resins [9,15] Polydimethylsiloxane (PDMS) is commonly used for replica molding in soft lithography due to its desirable properties such as flexibility, optical clarity, chemical inertness, and gas permeability [13]. Microfluidic devices fabricated by soft lithography are primarily planar with uniform height. Multiple steps are involved to fabricate non-planar 3D microchannels with varying heights [16]; non-planar microchannels are a requisite for applications such as droplet generation and manipulation [17,18,19,20,21], biological flow units [22,23], and soft robots [24]. For example, an additional dimension of non-planar channels permits the complex actuation of robotic structures; non-planar channels also allow handling fluids with different wettability without any modification to the surface. 3D printing has evolved as a rapid prototyping tool for 3D microchannels [25,26,27,28,29,30,31]. 3D printing can be employed to fabricate micromolds [28,29,30,31,32,33,34,35], sacrificial molds [36], and fluidic devices [37,38,39]. 3D printing of micromolds replaces the need for the photolithography used to fabricate a silicon master mold, which is adopted in the research community of microfluidics. In particular, stereolithography (SLA) printers and PolyJet 3D printers are suitable for creating molds for replica molding of PDMS and subsequent bonding with flat substrates [23,28,29,30,31,32,33,34]. Most of the commercially available 3D printers produce dimensions different from the manufacturer’s specification. There is a systematic deviation between the designed and printed dimensions, and the deviation varies with the types of printers. For instance, the minimum attainable dimension of the structure is ~200 μm for PolyJet printer, while the specified resolution is 42 μm (deviation is ~376%) [3,40]. In this work, we particularly focused on understanding this deviation observed for PolyJet printers.

### 1.3. PolyJet 3D Printing to Fabricate Micromolds

PolyJet printing has exhibited the potential to fabricate microscale structures including microfluidic molds [31,32,33]. PolyJet printing is a process to build 3D structures in layer-by-layer manners using photocurable polymeric ink. Owing to the smooth surface finish of the printed molds, PolyJet printing has been increasingly used to fabricate 3D molds for replica molding where the relief structures are printed on the mold [31,32,33]. The previous characterization of PolyJet printers revealed that the microstructures were fabricated with dimensions different from the design [1,2,3,22,23,33,34,35]. The printed structures were also found to exhibit leaning sides and rounded corners instead of straight lines and sharp corners [2,3,23,33]. For large structures (in centimeters), the discrepancy between the designed and printed dimensions may not be pronounced well relative to its original dimensions. However, change in dimensions and shapes of the microstructures (in sub-millimeter) result in large deviations affecting their usability. The discrepancy between the designed and the printed microstructures printed by PolyJet printers was attributed to uncontrolled reflow and the spreading of the resin [1,2,3]. A previous study on the characterization of PolyJet printers reported that the optimal width and height that can be produced was ~300 μm with a deviation >40% from designed geometry [23]. A comparison of four inkjet-based printers from Stratasys and Projet showed that all the printers produced structures with dimensions larger than the dimensions reported by the manufacturers [3]. This comparative work studied the variation in lateral dimension and devised a formula with correction factors to predict the printed width. These reports highlighted the deviation of the printed features in the lateral dimensions and predicted the expected width using linear relationships. However, practically, we should consider the contribution of both the designed dimensions (width and height) in achieving the printed dimensions. Indeed, the effect of the spreading of the resin on the vertical dimensions has not been examined. Understanding the fidelity in the printed height is particularly important when we fabricate molds for narrow microchannels with low aspect ratios. It is required to account for all the designed dimensions to develop a general predictive model for PolyJet printing.

To address this gap, this study revisited the characterization of the microstructures fabricated by PolyJet printer. A model PolyJet printer (Objet30 Prime™) was tested for its ability to fabricate cuboid-shaped microstructures with varying designed height (*h_d_*) and width (*w_d_*) (100–3000 μm). Crucially, we studied the effect of spreading on both the lateral and vertical dimensions. Our study revealed that the fabricated height of the microstructures was influenced by the designed width of the features. We found the threshold widths (*w_d_*′) below which the heights of the features were not printed as designed. In addition, we studied the effect of the layer thickness on the printing accuracy; we found that the layer thickness was also an important parameter to determine the degree of deviations in the width and height of the printed features. Further, we identified that the printer deposited excess resin in a particular mode of printing (High Speed (HS) mode; with layer thickness = 28 μm). In this mode, the height of the structures was not compromised as much as in other modes of printing. From the experimental observations, a mathematical model was developed to predict both the lateral and vertical dimensions of printed features of HS mode. The model was bifurcated to represent variations in printing in the regions of low and high widths. The accuracy of the model was tested by validating it with the experimental data. This study contributes to establishing a practical understanding of the inherent limitation of the printing mechanisms of PolyJet printing. Such an understanding shall be useful for the fabrication of microstructures by PolyJet 3D printers by identifying the design that could potentially offset the deformation of the microstructures.

## 2. Materials and Methods

### 2.1. Research Aims and Approach

This research aimed to characterize the dimensions of microscale features printed using a PolyJet 3D printer. The experiments included printing of cuboid-shaped microstructures designed with a wide range of *w_d_* and *h_d_* (100, 200, 300, 400, 500, 1500, and 3000 μm) and quantification of *w_p_* and *h_p_*; the subscript *d* denotes the designed dimension, and the subscript p denotes the printed dimension. The experiments were performed with a model PolyJet (Objet30 Prime) printer using Veroclear (an acrylate-based photoresin). Veroclear™ was suitable for fabricating microfluidic molds due to its rigidity and non-sticky surfaces after simple post-processing. Veroclear was used as a representative material of Vero™-based materials. Other Vero-based materials should exhibit a similar degree of deviation through the same mechanism as observed for Veroclear. PolyJet printers are a closed system that uses proprietary resins and settings for printing. We designed our study to characterize the printer in its original setting without any modification. Studying the commercial printer with its original settings and representative materials would be beneficial to gain an in-depth understanding of the printer’s performance. In this work, we studied the deviation between the designed and printed dimensions (width and height) in terms of (1) designed dimensions, and (2) the layer thickness of printing.

Firstly, the deviation between the designed dimensions (*w_d_* and *h_d_*) and the printed dimensions (*w_p_* and *h_p_*) was studied. The microscale structures were designed with rectangular cross-sections to observe the formation of curvatures after the spreading of the resin. While the effect of spreading on *w_p_* was discussed in detail in previous studies [1,2,3], detailed characterization on *h_p_* was not reported. Secondly, the influence of the layer thickness on the spreading of resin was tested. The layer thickness of the printing (preset by the printer in three different modes) determines the amount of resin deposited in each printing cycle. We hypothesized that the layer thickness would also influence the profile of spreading that affected the fidelity of printing. Importantly, the relationship between the designed and printed dimensions was studied to verify the hypothesis that the features need to be sufficiently wide to generate the designed height of the structures. Further, based on the experimental data, two mathematical models were developed to predict the printed dimensions for given designed dimensions. The models were developed to empirically represent the dynamics of PolyJet printing in the regions of low and high widths. For the low widths (below *w_d_*′), the height of the features failed to reach the intended height, and we observed a steady increase in *h_p_* with an increase in *w_d_*_._ Above *w_d_*′, *h_p_* attained *h_d_* and remained stagnant. To represent these two different behaviors, we used two separate quadratic models to fit the experimental data. Our models can serve as a practical guideline to predict the outcomes before printing.

### 2.2. Design of Microscale Features and PolyJet Printing

A 7 × 7 array of cuboid-shaped microstructures with *w_d_* and *h_d_* of 100, 200, 300, 400, 500, 1500 and 3000 μm were designed over a flat base using AutoCAD^®^ 2016 (Autodesk, CA, USA). The length of the cuboids was 1 cm and a gap of 0.5 cm was provided between each of the structures. The designed structures were 3D printed in Objet30 Prime™ (Stratasys, MN, USA) with Veroclear and support SUP705 as a model material and a support material, respectively. The structures were printed in three different layer thicknesses of 16, 28, and 36 μm, each of which is termed as high quality (HQ), high speed (HS), and Draft modes of Objet30 Prime printer, respectively. 

### 2.3. Post-Processing and Replica Molding

3D printed master molds were subjected to post-processing to remove the uncured resin and support material. Post-processing involved the removal of the support materials in a water jet followed by the soaking of the printed models in deionized water for 2 h. Afterward, the molds were baked in an oven at 60 °C for 24 h. The replica of the 3D printed mold was obtained by casting PDMS (Sylgard 184 Silicone Elastomer kit, Dow Corning, USA) mixed in a 10:1 ratio (by weight) of the prepolymer to the curing agent. Molds cast with PDMS were cured at 60 °C for 3 h. The cured PDMS replica was peeled off with the relief structures replicated from the master mold.

### 2.4. Characterization of Printed Features

To investigate the deviation between the designed and printed dimensions, cross-sections of the PDMS replica at three different places along the length of cuboid-shaped microchannels were taken. Precisely, the cross-sections were obtained from the center region ignoring the ends. The cross-sections were flipped to show the open rectangle and imaged using the MU500 AmScope (Irvine, CA, USA). The physical parameters (*w_p_*, *h_p_*, and cross-sectional area) were measured from the cross-sectional image using ImageJ (ImageJ, National Institutes of Health (NIH), Bethesda, MD, USA). The deviation in the dimensions between the designed and printed structures was examined with respect to the printing conditions (e.g., the designed dimensions and the layer thickness set by the printer).

### 2.5. Mathematical Modelling

Surface fitting of our experimental data was performed to predict the printed dimensions for HS mode using SciPy module in Python 3.8. We used a second-order polynomial to fit our data. The data represented two different regimes: (1) low widths (100, 200 μm) with *h_p_* less than the intended height and (2) high widths (300–3000 μm) where the intended height was printed. Owing to the two different behaviors observed, we used two different models to fit the data. The mathematical models predicted the dimensions of printed structures based on the input of designed dimensions. We validated the models against the experimental data and reported the accuracy of the models.

## 3. Results

### 3.1. Fabrication of Microstructures by PolyJet Printing

**Figure 1:**
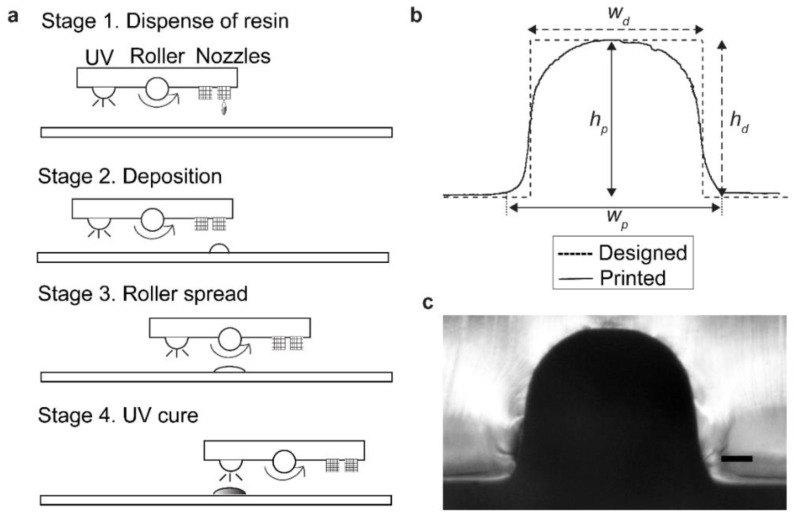
(**a**) Schematic illustration of the process of Polyjet printing. (**b**) Illustration showing the cross-sectional view of printed and designed structures. (**c**) Corresponding microscopic image obtained for the microscale structure with *w_d_* = *h_d_* = 500 μm. Scale bar = 100 μm.

We first fabricated samples with microstructures by Polyjet printing. PolyJet printing is derived from inkjet printing. Similar to printing inks, PolyJet uses photocurable polymer resins to form 3D models. Briefly, PolyJet printing is based on the following process: (1) deposition of an array of photopolymer droplets, (2) leveling of the deposited droplets by a heated roller, and (3) UV curing resulting in solidification of a single layer (Figure 1a). The process continued sequentially and repeatedly, and the 3D structures were built in a layer-by-layer manner. Figure 1b shows the overlaying sketch of the cross-section of designed and printed structures obtained from Objet30 Prime printer, and Figure 1c shows the microscopic image of the same feature (with w_d_ = h_d_ = 500 μm). We defined the maximum width (at the horizontal baseline) and the maximum height (at the vertical centerline) of the printed structures as the measurement for w_p_ and h_p_. The printed width (w_p_) deviated from the designed width (w_d_) by the spreading of the base that created curved corners. In contrast, in this example, the printed height (h_p_) was comparable to the designed height (h_d_) at the center of the feature. The printed cuboid attained a plateau at the top of the feature with a maximum height (that was comparable to h_d_). This observation led to the hypothesis that the feature must be sufficiently wide to attain the plateau with the designed height. We, therefore, studied the fidelity of printing for varying widths including narrow features. 

### 3.2. Deviation between the Designed and Printed Dimensions for HS Printing Mode

**Figure 2:**
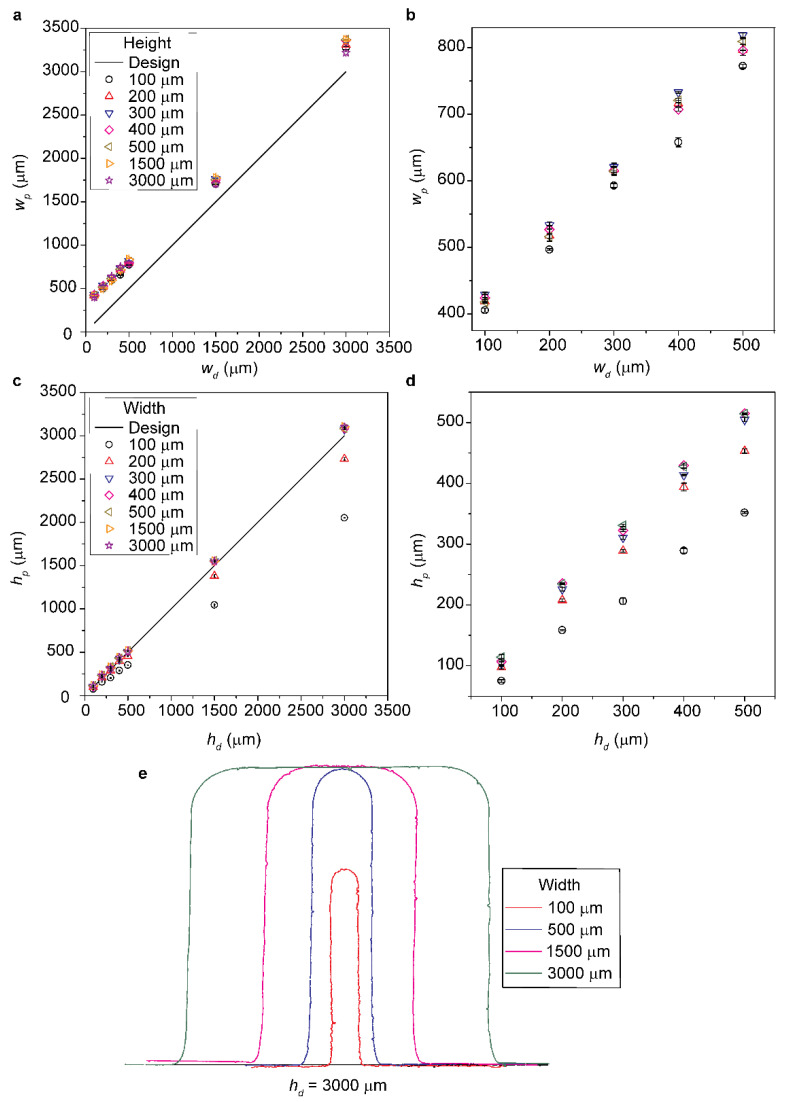
(**a**,**b**) Plot of wp against wd for *h_d_* = 100–3000 μm, and a magnified view for *w_d_* = 100–500 μm. (**c**,**d**) Plot of hp against hd for *w_d_* = 100–3000 μm, and a magnified view for *h_d_* = 100–500 μm. (**e**) Cross-sectional profile for *h_d_* = 3000 μm with varying wd, suggesting the decrease in the printed height for *w_d_* = 100 μm.

Objet30 Prime offers three preset modes of printing based on layer thickness; namely, HQ (16 μm), HS (28 μm), and Draft (36 μm). Initially, we used HS mode to evaluate the fidelity of the printing. The measurement showed that w_p_ was always greater than w_d_ for the range of the height we investigated (h_d_ = 100–3000 μm) (Figure 2a). The magnified view of w_p_ against w_d_ for 100–500 μm is shown (Figure 2b). This observation reconfirmed that spreading inevitably happened laterally in PolyJet printing [1,2]. The time lag between the deposition of resin and curing by UV led to the reflow of resin and spreading, which increased the lateral dimensions of the base of the printed features with curved corners (Figure 1c). We note that the extent of spreading of resin (and the resulting increase in the width) was the same for all the designed dimensions. Therefore, we reported the deviation with respect to their original values, not by the percentage. The report by the absolute values would provide a clear understanding of the mechanism that led to the deviation. 

We then investigated the height of the printed features. Figure 2c shows the plot of h_p_ against h_d_ for a range of w_d_. The magnified view of h_p_ against h_d_ for 100–500 μm is shown (Figure 2d). The measurement of h_p_ for HS mode showed that the actual height of the structures was not produced for w_d_ ≤ 200 μm. It is plausible that the resin deposited over the narrow width (w_d_ ≤ 200 μm) spread towards the base before UV curing without maintaining the volume of the resin to produce the intended height. The plot also suggested that the percentage of the decrease in the printed height was consistent for w_d_ = 100 μm (28.1% by average) and w_d_ = 200 μm (4.3% by average) regardless of h_d_, which was indicated by the linear trend for each series. This observation suggested the decrease in the printed height consistently occurred in each cycle of printing. For w_d_ ≥ 300 μm, the structures achieved the designed height (h_d_) at the center of the features. We observed that for w_d_ ≥ 300 μm, h_p_ obtained was slightly larger than h_d_ (~7.3% by average). This difference in h_p_ was systemic, and we attributed it to the calibration of the printer. Figure 2e shows the outline (i.e., upper surface) of the cross-section of the structures for varying w_d_ with the same height (h_d_ = 3000 μm). This illustration depicts that the structure printed with w_d_ = 100 μm was lower than the designed, while the structures with w_d_ ≥ 300 μm reached h_d_ at the centerline. The inability to produce designed h_p_ for low w_d_ suggested that PolyJet printer is not suitable to produce micromolds to replicate narrow channels.

### 3.3. Influence of Layer Thickness on the Spreading of Resin

In the previous section, we discussed that the spreading of the resin caused the deviation in the printed dimensions in HS mode (layer thickness = 28 μm). Albeit discretely, PolyJet printing offered different settings for layer thickness. To understand the influence of the layer thickness in the attainable microstructures, we studied the printed dimensions obtained in three available printing modes: HQ (16 μm), HS (28 μm), and Draft (36 μm). 

**Figure 3:**
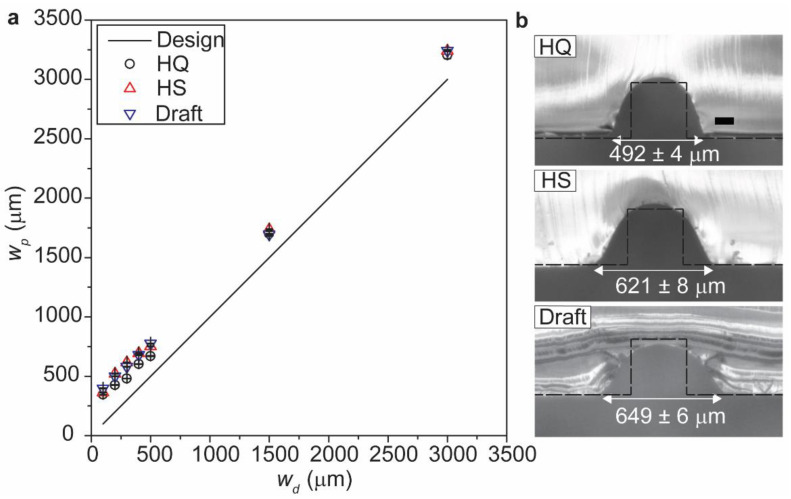
(**a**) Plot of the *w_p_* against w_d_ obtained for three different layer thickness: HQ (16 μm), HS (28 μm), and Draft (36 μm). (**b**) Microscopic images showing the cross-section of the designed and printed cuboid-shaped structures replicated in PDMS for the three different modes of printing. The microstructures were with *w_d_* = *h_d_* = 300 μm. Scale bar = 100 μm.

The plot of w_p_ against w_d_ depicts the deviation in dimensions of the printed structures from the designed dimensions (Figure 3a). The spreading of resin was more pronounced with the larger layer thickness due to the higher volume of resin deposited in each cycle. HQ mode (with the lowest layer thickness) exhibited the least deviation in dimension among the three modes investigated. Therefore, we concluded that layer thickness is a major factor that decided the extent of spreading of resin. Figure 3b shows the cross-sectional views at the center of the designed and the printed structures (replicated with PDMS) with w_d_ = h_d_ = 300 μm. We observed that w_p_ of the structures exceeded w_d_ for all the layer thickness, while the degree of spreading increased in the order of the layer height. Interestingly, however, the spreading of the resin for HS and Draft modes are comparable despite the difference in the layer heights (28 μm and 36 μm). We discuss this observation in the later sections by analyzing the volume of the resins deposited in each mode of printing.

### 3.4. Characterization of Printed Height for Varying Designed Widths

**Figure 4:**
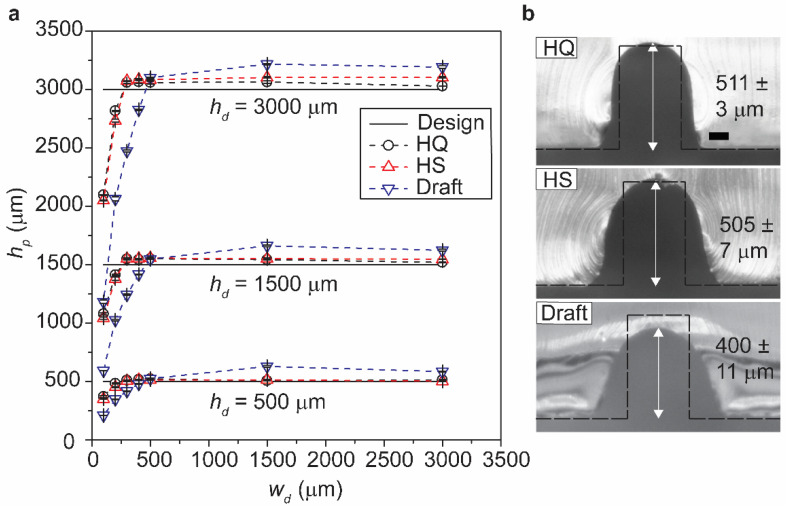
(**a**) Plot of *h_p_* against *w_d_* for the three different layer thickness: HQ (16 μm), HS (28 μm), and Draft (36 μm) for *h_d_* = 500, 1500 and 3000 μm. HQ and HS modes required *w_d_* = 300 μm to achieve *h_p_* > *h_d_*, while Draft mode required *w_d_* = 500 μm to achieve *h_p_* > *h_d_*. (**b**) Microscopic images showing the cross-section of designed and printed cuboid-shaped structures replicated in PDMS for the three different modes of printing. The microstructures were with *w_d_* = 300 μm and *h_d_* = 500 μm. Scale bar = 100 μm.

The previous section discussed the influence of the layer thickness on the width of the printed features (w_p_). We also studied the effect of the layer thickness on the height of the printed features (h_p_) for varying designed widths (w_d_). The plot shows h_p_ of the structures obtained for h_d_ = 500, 1500, and 3000 μm against varying w_d_ (Figure 4a). As discussed earlier, h_d_ was attained only when the structures were designed above a particular threshold (w_d_’). From the measurement of h_p_, w_d_’ was identified to be 300 μm for HQ and HS modes, and 500 μm for Draft mode, respectively. Figure 4b depicts the cross-section of the designed and printed structures (replicated with PDMS) taken at the center of the microchannel. For w_d_ = 300 μm, h_d_ was attained in HQ and HS modes but not in Draft mode. These observations suggested that layer thickness was another key parameter (in addition to the dimension of the features) to determine the fidelity of printing in terms of both width and height. While the layer height was preset by the printer at discrete values, decreasing the layer height resulted in decreasing the lateral spreading (that increased the print fidelity in width) and the threshold width (w_d_’) (that increased the print fidelity in height). 

### 3.5. Fabrication of Intended Height by HS Printing Mode

**Figure 5:**
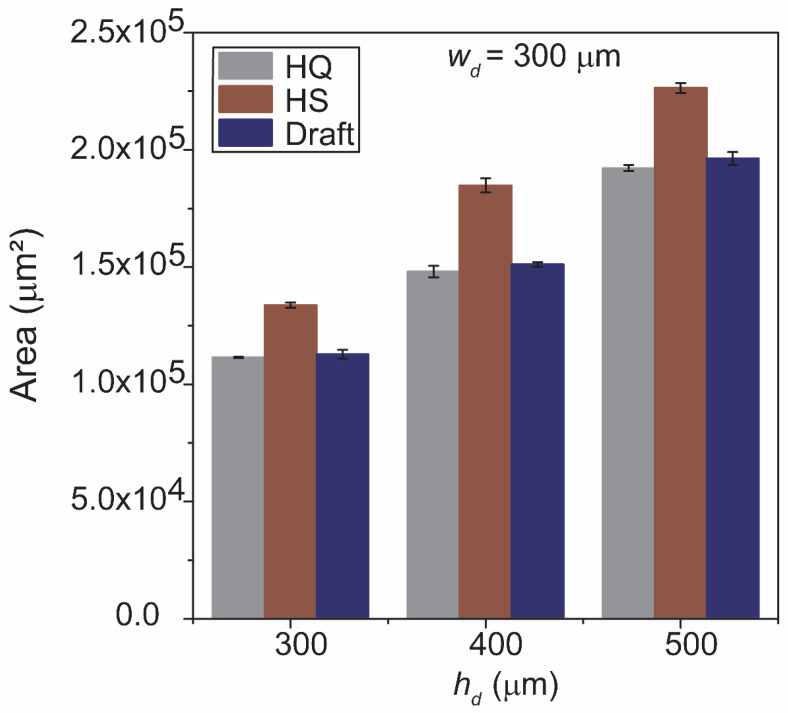
Bar graph showing the cross-sectional area of the structures obtained for the three different layer thickness: HQ (16 μm), HS (28 μm) and Draft (36 μm). The features were with *w_d_* = 300 μm and *h_d_* = 300, 400 and 500 μm.

Our original hypothesis was that HS mode (with the intermediate layer thickness) would exhibit the degree of spreading between HQ and Draft modes. However, our study of the printed structures provided conflicting observations; Figure 3b suggested that the degree of spreading was similar between HS and Draft modes, while Figure 4a suggested the threshold width (w_d_’) was the same for HS and HQ modes. To explain these observations, we measured the cross-sectional area of the printed structures (which were the indication of the deposited volume of the resin) obtained for three printing modes. Figure 5 shows a bar graph of the cross-sectional area of the structures with w_d_ = 300 μm and h_d_ = 300, 400, and 500 μm. The graph revealed that, with the same design, the structures printed in HS mode were systemically larger (i.e., a higher volume of the resin was deposited) than the structures printed in HQ and Draft modes. Based on the image analysis, we concluded that HS mode deposited a larger amount of resin (by ~16.5% by average) than the other two modes. The additional resin in HS mode contributed to the lateral spreading of the resin equivalent to Draft mode, while it allowed printing the designed height for narrow features by maintaining a sufficient volume of resins after spreading.

The available print modes are predefined and cannot be altered. However, our experiment implied that the volume of the resin printed in each cycle is another key parameter to achieve intended microstructures. The increase in the resin volume, in principle, helps to achieve the intended height of the microstructures with the trade-off for the increased width of the printed features. Such understanding would help to calibrate the printer when open-source polymer-jetting printers become available.

### 3.6. Mathematical Modelling

**Figure 6:**
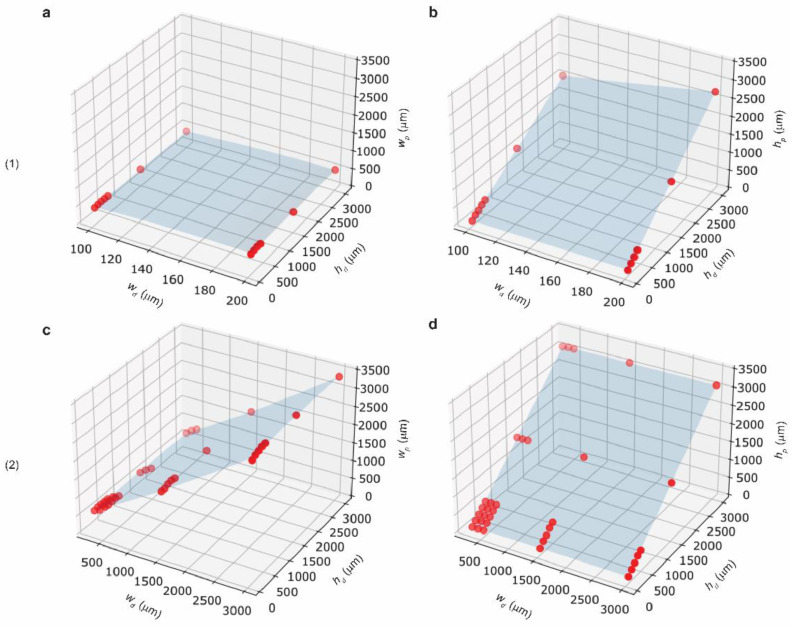
Plots showing the surface fitting of experimental data of HS mode. (**a**,**b**) represent the modeled surface for *w_p_* and *h_p_* of low widths. (**c**,**d**) represent the modeled surface for *w_p_* and *h_p_* of high widths.

Lastly, we fitted our experimental data obtained for HS mode on a surface using second-order polynomials. We developed the models for two cases—(1) region of low widths (100 μm, 200 μm) where the intended height was not achieved and (2) region of high widths (300–3000 μm) that was produced with intended height. Polynomial equations were obtained for the printed width and height of the two regimes of HS mode. The model equation is given as:

(1) $$Z\left(x,y\right)=\;C_{4}x^{2}+\;C_{5}y^{2}+C_{3}xy+\;C_{1}x+\;C_{2}y+C_{0}$$

where x and y denote the designed width and height, respectively. Z(x,y) represent printed width or height estimated for the given designed width and height. Parameters of the model are tabulated (Table 1). Plots describing the fit of the experimental data with the modeled surfaces are provided (Figure 6). The coefficient of determination ($R^{2}$) gives the value of how close the experimental data lies to the modeled surface. We obtained a $R^{2}$ value > 96% for all our model equations. We tested the dimensions of 200 μm × 500 μm and 900 μm × 900 μm (*w_d_* × *h_d_*) (called Case (1) and Case (2), respectively). The tested dimensions were excluded during the development of the model. The model predicted *h_p_* < *h_d_* in Case (1) and *h_p_*~*h_d_* in Case (2). The observation suggested that our model efficiently represented the actual feature dimensions of PolyJet printing. The predicted values of the two cases matched closely with the experimental data. The deviation between the predicted dimensions and experimental measurement was in the range of 0.1–4.5%. Thus, our model can be employed to predict the outcomes of PolyJet printing. Using this model, one can estimate the printed dimensions beforehand and account for appropriate corrections to the designed dimensions.

## 4. Conclusions

This paper discussed the characterization of the lateral and vertical dimensions of the cuboid-shaped microstructures printed by Objet30 Prime 3D printer. We made the following key observations that were not reported in the previous studies: (1) the spreading of resin affected both *w_p_* and *h_p_* of the structures; (2) the printed structures attained *h_d_* only when *w_d_* was above a particular threshold width (*w_d_*′); (3) the layer thickness of printing was an important parameter to determine *w_p_* and *h_p_*; (4) an excess resin in each printing cycle allowed achieving an intended height with the trade-off for the increased width by spreading in HS mode. The fidelity of printing is determined by both the lateral and the vertical dimensions of the designed structures. The threshold width (*w_d_*′) to ensure the fidelity of the height was 300 μm for HQ and HS modes and 500 μm for Draft mode. Interestingly, our analysis suggested that HS mode deposited additional resin in each cycle of printing, which helped in forming the intended height while exhibiting spreading equivalent to Draft mode. In addition to the above experimental findings, we developed two mathematical models to predict the outcomes of PolyJet printing for the regions of low and high widths of HS mode. The models successfully predicted both the lateral and vertical dimensions based on the designed dimensions. The models represented the experimental data in the closeness of $R^{2}$ > 96%. Validation of the models showed that the maximum deviation was less than 4.5% for both regimes. The prediction that the printed height did not reach the intended height for low-width features was a crucial contribution of our work. Our study would serve as a guideline to choose the appropriate dimensions of the features and layer thickness of printing to fabricate features with required fidelity.

This study contributes to establishing a practical understanding of the inherent limitations of PolyJet printers. The outcomes of our work would be beneficial to comprehend the influencing parameters of the printing. PolyJet printers are increasingly employed for fabricating molds for non-planar microfluidic devices. At present, the limitation of the resolution of printed microstructures prevents PolyJet printers to fabricate microstructures with high fidelity. The findings of our work can help in designing microstructures under the limitation of PolyJet printers, which can be useful in microfluidics, soft robotics, and other fields of engineering employing replica molding based on 3D-printed micromolds.

## Figures and Tables

**Table 1:** Parameters of the mathematical model to predict printed dimensions.

Printed Dimension	$R^{2}$	$C_{0}$	$C_{1}$	$C_{2}$	$C_{3}$	$C_{4}$	$C_{5}$
*w_p_* (1)	0.9632	8.76 × 10^−2^	5.84	−1.78 × 10^−2^	8.77 × 10^−5^	−1.63 × 10^−2^	4.58 × 10^−7^
*h_p_* (1)	0.9999	1.23 × 10^−3^	8.21 × 10^−2^	4.73 × 10^−1^	2.25 × 10^−3^	−7.77 × 10^−5^	−5.57 × 10^−6^
*w_p_* (2)	0.9995	3.41 × 10^2^	8.54 × 10^−1^	3.94 × 10^−2^	7.62 × 10^−6^	4.22 × 10^−5^	−1.00 × 10^−5^
*h_p_* (2)	0.9999	6.58	1.62 × 10^−1^	1.02	1.68 × 10^−6^	−4.45 × 10^−6^	1.49 × 10^−6^
